# A Temperature-to-Frequency Converter-Based On-Chip Temperature Sensor with an Inaccuracy of +0.65 °C/−0.49 °C

**DOI:** 10.3390/s23115169

**Published:** 2023-05-29

**Authors:** Zicong Xu, Xuan Zhang, Sili Chen, Jiahao Cheong, Lei Yao

**Affiliations:** 1 School of Microelectronics, Shanghai University, Shanghai 201800, China; 2 The Shanghai Industrial μTechnology Research Institute, Shanghai 201899, China

**Keywords:** temperature sensor, relaxation oscillator, frequency divider, two-point calibration

## Abstract

This paper proposes a temperature sensor based on temperature-frequency conversion using 180 nm CMOS technology. The temperature sensor consists of a proportional-to-absolute temperature (PTAT) current generating circuit, a relaxation oscillator with oscillation frequency proportional to temperature (OSC-PTAT), a relaxation oscillator with oscillation frequency independent of temperature (OSC-CON), and a divider circuit cascaded with D flip-flops. Using BJT as the temperature sensing module, the sensor has the advantages of high accuracy and high resolution. An oscillator that uses PTAT current to charge and discharge capacitors to achieve oscillation, and utilizes voltage average feedback (VAF) to enhance the frequency stability of the oscillator is tested. Through the dual temperature sensing process with the same structure, the influence of variables such as power supply voltage, device, and process deviation can be reduced to a certain extent. The temperature sensor in this paper was implemented and tested with a temperature measurement range of 0–100 °C, an inaccuracy of +0.65 °C/−0.49 °C after two-point calibration, a resolution of 0.003 °C, a resolution Figure of Merit (FOM) of 6.7 pJ/K^2^, an area of 0.059 mm^2^, and a power consumption of 32.9 μW.

## 1. Introduction

Over the past 50 years, the semiconductor industry has followed Moore’s Law [1]. With advances in process technology, the device size of circuits has continuously decreased, resulting in increased integration density of circuits within chips. The power density inside chips has also increased, leading to a rise in temperature during normal operation that affects the performance of the circuits [2]. Failure to dissipate heat normally can cause irreversible damage to the chip. Therefore, it is essential to incorporate temperature sensors into chips to monitor their temperature in real time [3].

Different types of sensors have their advantages and disadvantages, and the most suitable temperature sensor should be selected according to different application scenarios. Depending on the temperature sensing device, CMOS temperature sensors can be divided into BJT-type, resistor-type, full MOS transistor-type, and thermal diffusivity (TD) -type [4,5,6,7]. Based on the current mainstream architecture, on-chip integrated CMOS temperature sensors can be roughly divided into two categories: voltage-domain and time-domain temperature sensors. In brief, voltage-domain temperature sensors convert temperature into a corresponding voltage value using their temperature sensing module, and an analog-to-digital converter (ADC) is generally used to convert the voltage signal to a digital signal to transition from temperature to digital output. The specific selection of the ADC type generally depends on the temperature sensing module and system indicators. Time domain temperature sensors convert the voltage or current signal generated by the temperature sensing module into the time domain for processing, such as converting it into temperature-related frequency, period, or duty cycle, and then selecting the appropriate readout circuit to output digital encoding [8]. The different architectures lead to differences in the selection of the analog-to-digital conversion circuit, which directly affects the performance differences of CMOS temperature sensors. Voltage-domain CMOS temperature sensors often face problems such as complex structure, large area, and high power consumption due to the selection of ADCs. However, their accuracy is generally high. On the other hand, time-domain temperature sensors—as they select digital circuits such as time-to-digital converter (TDC) or frequency-to-digital converter (FDC) [9,10]—can often build most circuits using standard cell libraries, and have strong adaptability, simple structure, small area, and low power consumption. Therefore, it is necessary to determine CMOS temperature sensors with different architectures in advance according to different needs.

This article presents an on-chip temperature sensor with a temperature-frequency conversion mode, which can reduce the impact of some interference variables such as power supply voltage, device, and process deviations to a certain extent through a twin-temperature sensing process architecture with the same structure [11]. Based on the time domain, CMOS temperature sensors have a low accuracy and are susceptible to power supply voltage disturbances if external modules such as Low-dropout regulators (LDO) are not used due to their structure. Therefore, this architecture is one of the solutions to address the poor PV (Process-Voltage) characteristics of time-domain CMOS temperature sensors. The temperature sensor can achieve a temperature measurement range of 0–100 °C and, after a two-point calibration, the inaccuracy is +0.65 °C/−0.49 °C, the resolution is 0.003 °C, the area is 0.059 mm^2^, and the power consumption is 32.9 μW.

Section 1 introduces the type and application environment of on-chip temperature sensors and the advantages of this temperature sensor. Section 2 describes the circuit design principle of the temperature sensor. Section 3 presents the simulation results using simulation tools. Section 4 presents the test results after chip implementation. Finally, Section 5 provides a summary and conclusion.

## 2. Circuit Design of Temperature Sensor

### 2.1. Top-Level Architecture 

**Figure 1:**
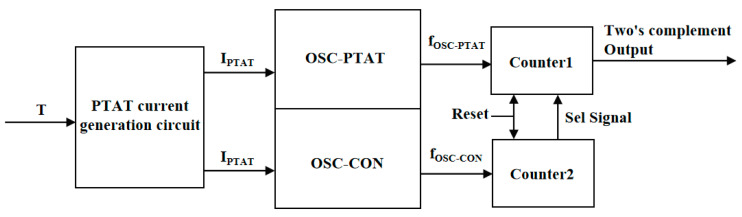
Block diagram of the temperature sensing principle of the temperature sensor.

Figure 1 depicts a block diagram of the on-chip temperature sensor principle. The temperature sensor consists of a current generating circuit that is proportional to the absolute temperature, a relaxation oscillator with oscillation frequency proportional to temperature (OSC-PTAT), a relaxation oscillator with oscillation frequency independent of temperature (OSC-CON), and a frequency-to-digital converter circuit. The PTAT current is generated by the temperature sensing circuit and used to charge the OSC-CON and OSC-PTAT modules. The OSC-CON module produces a reference clock independent of temperature, while the OSC-PTAT module generates a clock signal with frequency proportional to temperature. Two digital converters count the signals generated by the two modules, respectively. Both frequency-to-digital converters are controlled by an enable signal for simultaneous counting. When the digital converter of the OSC-CON module reaches its maximum count, it stops counting and triggers the digital converter of the OSC-PTAT module to stop counting as well. At this point, the OSC-PTAT outputs a binary code word proportional to temperature.

### 2.2. PTAT Current Circuit

**Figure 2:**
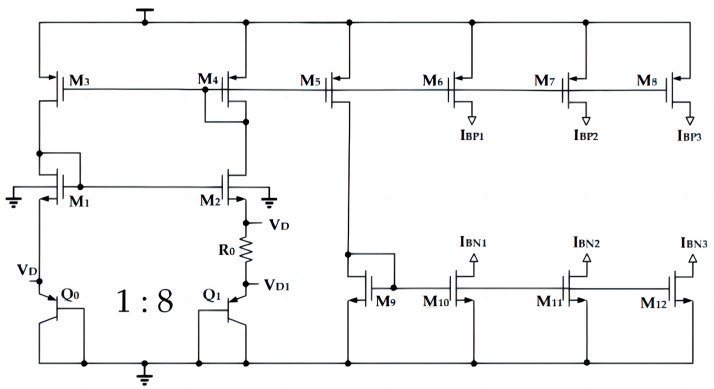
PTAT current circuit of temperature sensor.

The temperature sensing circuit transmits temperature by observing the characteristic of the junction voltage of BJT with temperature variation. Using BJT as the temperature sensing module has the advantages of high precision and high resolution. As shown in Figure 2, PNP triodes, $Q_{0}$ and $Q_{1},$ are connected as diodes, because the number of parallel transistors in two PNP transistors is different, the voltage on $Q_{0}$ is $V_{D}$, the voltage on $Q_{1}$ is $V_{D1}$, and the voltage on resistor $R_{0}$ is the voltage difference of two PNP transistors, $\Delta V_{BE}$, which can be calculated by Equation (2). It is a voltage proportional to temperature, and $\Delta V_{BE}$ divided by $R_{0}$ yields a current proportional to temperature [12]. Table 1 shows the CMOS parameters of the PTAT current generation circuit.

(1) $$V_{T}=\frac{kT}{q}$$

(2) $${\Delta V}_{BE}=V_{D}-V_{D1}=V_{T}\ln\left(\frac{I_{c}}{I_{s}}\right)-V_{T}\ln\left(\frac{I_{c}}{{nI}_{s}}\right)=V_{T}\ln\left(n\right)=\frac{kT}{q}\ln\left(n\right)$$

(3) $$I_{PTAT}=\frac{kT}{qR_{0}}\ln\left(n\right)$$

Here, *q* represents the electronic charge, *k* is the Boltzmann constant, *I_C_* and *I_S_* are, respectively, the collector and saturation currents of the PNP, *T* is the absolute temperature, and n represents the number of transistors in parallel with $Q_{1}$. In order to achieve circuit matching, the circuit in this paper was designed with n = 8.

### 2.3. Principles of OSC Circuit Design

Common oscillation methods include crystal oscillators, traditional RC oscillators, traditional oscillators that use PTAT current to charge and discharge capacitors for oscillation, bandgap-reference ring oscillators, bandgap-reference relaxation oscillators, and hybrid oscillators with peak-holding feedback of both relaxation and ring types [13,14,15,16,17]. However, all of the aforementioned oscillation methods have certain drawbacks. The frequency variation of waveforms generated by crystal oscillators is on the order of ppm, but they are costly and cannot be integrated into a chip. The oscillation frequency variation caused by the circuit structure of traditional RC oscillators, traditional oscillators that use PTAT current to charge and discharge capacitors for oscillation, bandgap-reference ring oscillators, bandgap-reference relaxation oscillators, and hybrid oscillators with peak-holding feedback of both relaxation and ring types exceeds ±1% with changes in voltage and temperature.

**Figure 3:**
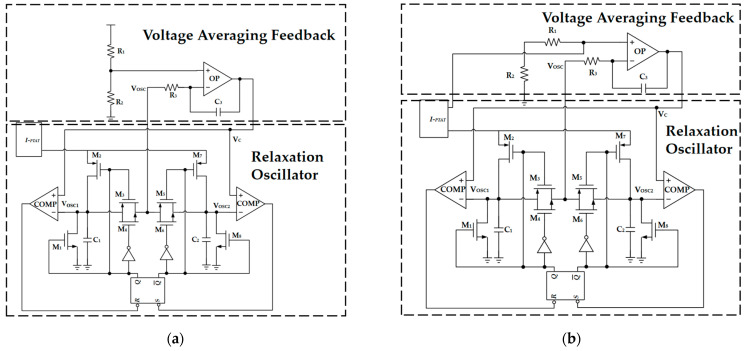
The circuit diagrams of relaxation oscillators. (**a**) The circuit diagram of a relaxation oscillator with oscillation frequency proportional to temperature (OSC-PTAT). (**b**) The circuit diagram of a relaxation oscillator with oscillation frequency independent of temperature (OSC-CON).

In traditional oscillator circuits that use PTAT current to charge and discharge capacitors to achieve oscillation, variations in the delay $t_{d}$ of comparators and RS flip-flops result in frequency changes with respect to voltage and temperature. The aging of current sources can degrade the accuracy of the $V_{osc}$ slope and cause frequency changes. The flicker noise of current sources accumulates jitter. This paper introduces the concept of Voltage Average Feedback (VAF) [18], proposes oscillators’ structure with VAF, and implements oscillation by charging and discharging capacitors with PTAT current—as shown in Figure 3—to adjust the output voltage $V_{C}$ of the VAF circuit based on the size of the delay time $t_{d}$ of comparators and RS flip-flops, thereby achieving oscillator stability independent of comparator and RS flip-flop delays, and eliminating cumulative jitter in the circuit. When the temperature varies from 0 to 100 °C, the output frequency of the oscillator changes by 0.08%, thereby improving the frequency stability of the relaxation oscillator.

Taking OSC-PTAT as an example, the mechanism of the oscillation waveform generation is described as shown in Figure 3a: Assuming that the RS trigger composed of the NAND gate is in the reset state, when *R* = “*1*” and *S* = “*0*”, *Q* outputs “*0*” and *Q*’ outputs “*1*“, *M*_1_, *M*_5_, *M*_6_, and *M*_7_ are cut off while *M*_2_, *M*_3_, *M*_4_, and *M*_8_ are turned on, and the left comparator circuit of the circuit works. The current source charges capacitor *C*_1_, causing $V_{osc1}$ to rise, and $V_{osc1}$ is transmitted as $V_{osc}$ to the reverse input of the VAF circuit. The right comparator circuit of the circuit does not work, and the $V_{osc2}$ output is 0 V. At the same time, when the circuit starts to work, the active filter composed of *R*_3_ and *C*_3_ makes the output voltage $V_{C}$ a constant value. When $V_{osc1}$ exceeds $V_{C}$, the left comparator outputs “*0*”, while the right comparator still outputs “*1*”. At this time, *R* = “*0*” and *S* = “*1*”, the RS trigger is in the set state, *Q* outputs “*1*”, *Q*’ outputs “*0*”, *M*_1_, *M*_5_, *M*_6_, and *M*_7_ are turned on while *M*_2_, *M*_3_, *M*_4_, and *M*_8_ are cut off, and the right comparator circuit of the circuit works. The current source charges capacitor *C*_2_, causing $V_{osc2}$ to rise, and $V_{osc2}$ is transmitted as $V_{osc}$ to the negative input of the VAF circuit. The left comparator circuit of the circuit does not work, and the $V_{osc1}$ output is 0 V. When $V_{osc2}$ exceeds $V_{C}$, the RS trigger returns to the reset state. Therefore, the RS trigger is always in the alternating state of reset and set, and the waveforms of $V_{osc1}$ and $V_{osc2}$ alternately transmit to the reverse output of the VAF circuit, thereby generating a stable oscillation waveform. Similarly, the oscillation mechanism of OSC-CON is roughly the same as that of OSC-PTAT, so it is not repeated here.

Temperature-to-frequency conversion in OSC:

(4) $$\frac{1}{T/2}\int_{0}^{\frac{T}{2}}\frac{I_{PTAT}\cdot t}{C_{1}}dt=V_{ref}$$

(5) $$f=\frac{I_{PTAT}}{4V_{ref}C_{1}}$$

By substituting Equation (3) into Equation (5), the following expression is obtained:

(6) $$f=\frac{kT\ln\left(n\right)}{qRCV_{ref}}$$

As shown in Figure 3a, $V_{ref}=R_{2}{\cdot V}_{dd}/\left(R_{1}+R_{2}\right)$, which can be substituted into the above equation to obtain a signal oscillation frequency that is directly proportional to the temperature for the OSC-PTAT circuit. As shown in Figure 3b, $V_{ref}=\left(R_{1}+R_{2}\right)\cdot I_{PTAT}$, which can be substituted into the above equation to obtain a signal oscillation frequency that is independent of temperature for the OSC-CON circuit.

**Figure 4:**
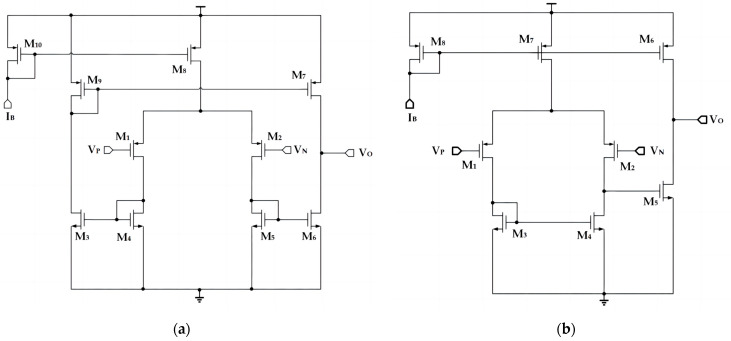
Operational amplifiers and comparators in the OSC-PTAT and OSC-CON circuits. (**a**) Circuit diagram of the operational amplifier. (**b**) Circuit diagram of the comparator.

The circuit diagrams of the operational amplifier and the comparator in both the OSC-PTAT and OSC-CON circuits are presented in Figure 4a and b, respectively. The operational amplifier uses a symmetric OTA (Operational Transconductance Amplifier) architecture, providing higher bandwidth. The comparator uses a two-stage operational amplifier structure, offering elevated DC gain. Table 2 and Table 3 show the parameters of the CMOS transistors in operational amplifiers and some performance indicators of operational amplifiers, and the parameters of the CMOS transistors in comparators and some performance indicators of comparators, respectively.

### 2.4. Frequency-to-Digital Converter

**Figure 5:**
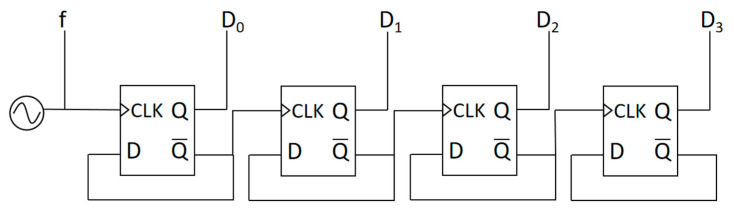
Cascaded D flip-flops are used to implement circuit counting.

The square wave signals generated by OSC-PTAT and OSC-CON are, respectively, input into cascaded D flip-flops shown in Figure 5 as the clock signal of the first D flip-flop. When a clock pulse arrives, the first D flip-flop sends the D input data to the *Q* output and the inverted data of *Q* to the *Q*’ output, which serves as the clock signal of the next stage D flip-flop. The same process is repeated for the next clock pulse, but with the input data already inverted. This results in the *Q* output changing its state every two clock pulses, achieving a halving of the frequency. By cascading D flip-flops, a frequency divider circuit is implemented which converts the signal frequency into a binary counting function. The counting value of OSC-CON is taken as a reference to observe the counting value of OSC-PTAT at this time.

The designed counter in this paper is 16-bit, and a 4-bit counter is taken as an example to explain the principle of circuit counting. The square wave signals generated by OSC-CON and OSC-PTAT are input into the digital frequency converter and the outputs are DC [0]-DC [3] and DP [0]-DP [3], respectively. Since the output frequency of the OSC-CON signal is independent of temperature, the counting time is fixed when the number of counting bits is determined. The 4-bit overflow value of DC [0]-DC [3] is taken as the counting reference to observe the counting value of DP [0]-DP [3]. DP is output in 4-bit binary complement form, and its value is exactly the difference between the two counters. Finally, the corresponding decimal number of DP [0]-DP [3] and the temperature are in a first-order linear relationship.

## 3. Simulation Results

**Figure 6:**
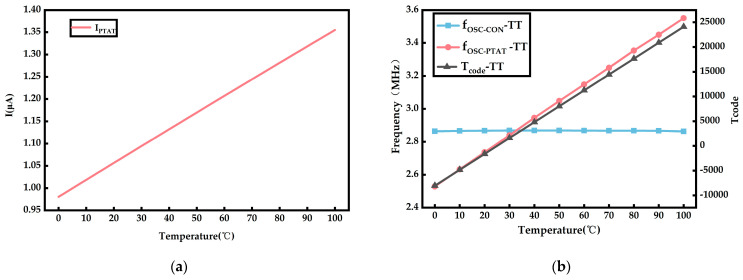
Under the TT process corner, the simulation results of the circuit when temperature varies from 0 to 100 °C. (**a**) Graph of the relationship between output current of the bias circuit and temperature. (**b**) The variation of $f_{OSC-CON}$, $f_{OSC-PTAT}$, and $T_{code}$ with temperature.

The circuit design was implemented using the SMIC 180 nm mixed signal CMOS technology in Cadence 6.17, and the circuit was simulated using the spectre tool. Under the TT process corner, Figure 6a shows that the PTAT current output of the temperature sensing circuit is proportional to the temperature ranging from 0 °C to 100 °C. Figure 6b shows that, as the temperature varies from 0 °C to 100 °C, the frequency of $f_{OSC-CON}$ remains relatively constant, while the frequency of $f_{OSC-PTAT}$ varies in direct proportion to the temperature. The output code $T_{code}$ is proportional to the temperature.

**Figure 7:**
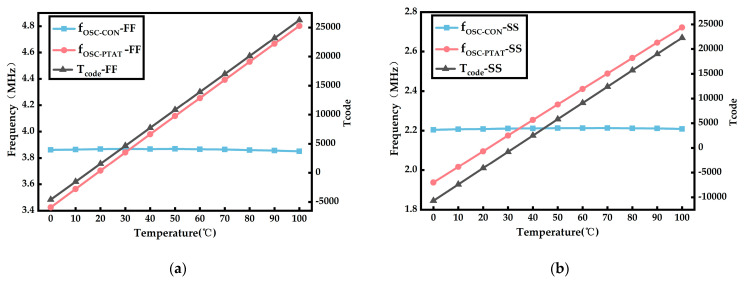
Variation of parameters at different process angles. (**a**) Under the FF process corner. (**b**) Under the SS process corner.

As shown in Figure 7, the outputs of $f_{OSC-CON}$, $f_{OSC-PTAT}$, and $T_{code}$ change as the temperature changes from 0 °C to 100 °C for the FF and SS process corners.

**Figure 8:**
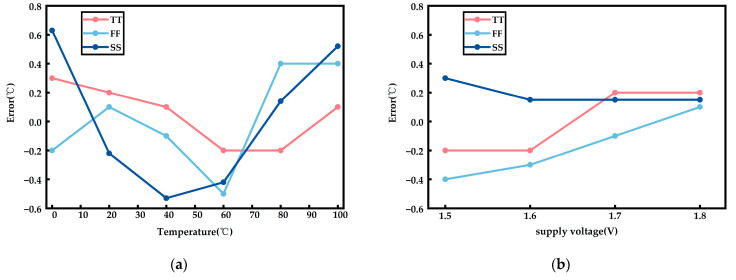
The temperature measurement error of the temperature sensor. (**a**) The temperature error curves of the temperature sensor under various corners. (**b**) At 40 °C, the temperature measurement error of the temperature sensor under various corners.

As shown in Figure 8, the temperature measurement error of the temperature sensor is evaluated when the temperature is varied from 0 to 100 °C under different process corners; namely TT, SS, and FF. At 40 °C, the temperature measurement error of the temperature sensor under various corners varies at different supply voltages of 1.5–1.8 V.

**Figure 9:**
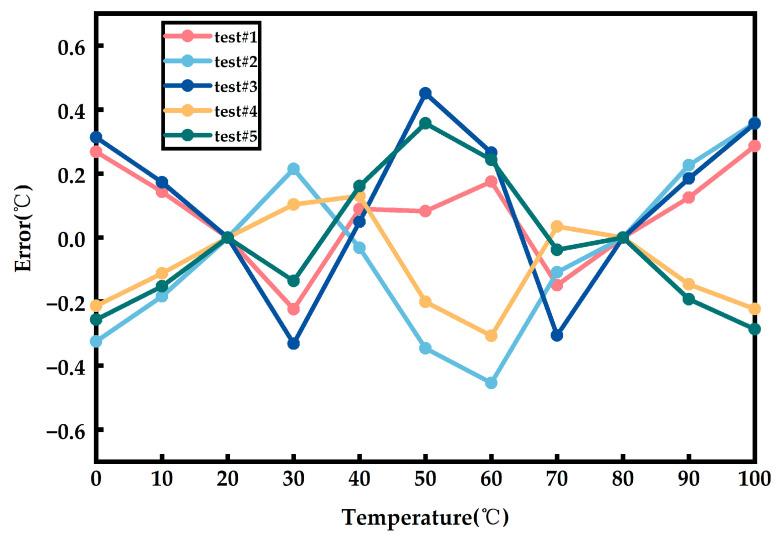
The Monte Carlo simulation temperature error after two-point calibration.

The circuit is subjected to five Monte Carlo simulations with temperature information added under the TT process corner. The result after two-point calibration is shown in Figure 9.

## 4. Test Results

**Figure 10:**
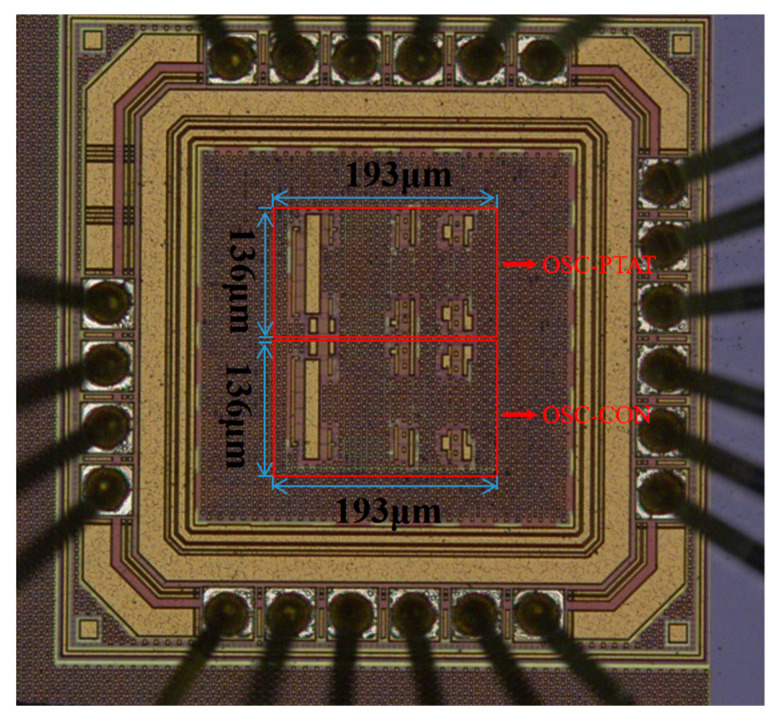
Microphotograph of the temperature sensor chip.

The on-chip temperature sensor proposed in this paper was designed and implemented in SMIC standard 180 nm CMOS process. The core area of the circuit was 0.059 mm^2^, as shown in the microphotograph in Figure 10.

**Figure 11:**
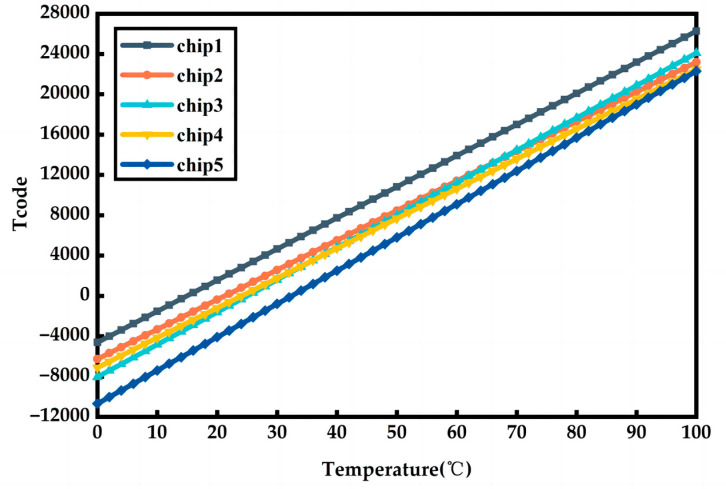
The temperature and output code of five test chips.

Five test chips were packaged in QFN and tested in high and low temperature chambers. Among them, DS18B20 was used for chip reference temperature measurement to obtain sufficient accuracy of the temperature of the chip environment. At the same time, a single-chip microcontroller development board was used to obtain and record the output code words. The operating voltage was 1.5 V, the temperature measurement range was 0–100 °C, and the temperature measurement step was 10 °C. Figure 11 shows the temperature of the test signal and the final output code word $T_{code}$ for the five chips. The output frequency of the chip test is linearly related to the temperature, and the output code word range is approximately −10688 to 26266 for a temperature range of 0–100 °C, corresponding to a resolution of 0.003 °C.

**Figure 12:**
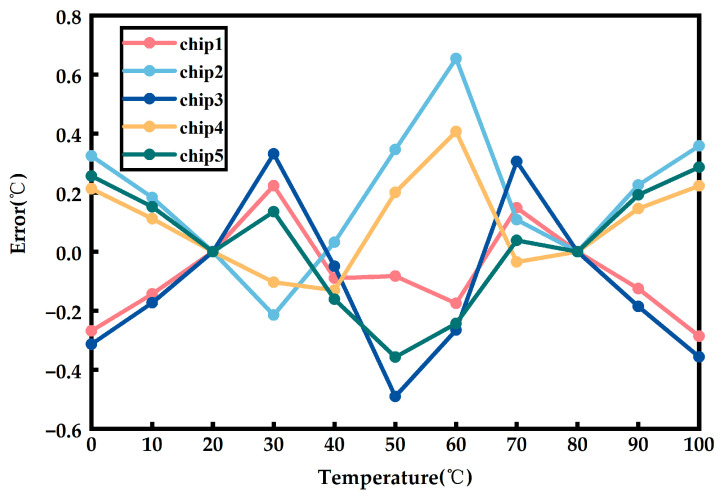
Temperature measurement error of the chip after two-point calibration.

As shown in Figure 12, five temperature sensor chips were tested. The final output code $T_{code}$ is a function of temperature: $T_{code}=f(T)$. Then, the output signal amplitude under any two temperature points ($T_{0}$, $T_{1}$) is determined, assuming that the temperature measurement error $\Delta T_{Error}$ is 0 at these two points. This determines the coordinates of the two points, which are used to obtain the calibrated “ideal curve”. Finally, the temperature measurement error at each point can be obtained based on the definition of temperature measurement error. By performing two-point calibration, the temperature measurement error is within +0.65 °C/−0.49 °C.

Table 4 summarizes the performance parameters of the CMOS temperature sensor designed in this paper and compares them with the performance of other sensors. 

## 5. Conclusions

In this paper, we have presented an on-chip temperature sensor based on temperature-frequency conversion for real-time temperature monitoring of the chip. By using BJT for temperature sensing, VAF for debouncing, and a dual-sensing process architecture, the impact of variables such as power supply voltage, device, and process deviations have been reduced, generating a stable oscillation frequency. Finally, the signal frequency is converted into binary numbers by a frequency-to-digital converter. The temperature sensor was tested after being fabricated using a 180 nm CMOS process with a temperature measurement range of 0°C–100°C, a resolution of 0.003°C, an area of 0.465 mm^2^, a power consumption of 32.9 μW, a conversion time of 22.75 ms, a resolution of 0.003 °C, a resolution FOM of 6.7 pJ/K^2^, and an accuracy after two-point calibration of +0.65 °C/−0.49 °C. The proposed design has addressed the problem of low accuracy in traditional temperature-frequency conversion-based temperature sensors.

## Figures and Tables

**Table 1:** Parameters of the CMOS transistors in the PTAT current circuit.

CMOS Parameters			
Instance Name	W (μm)/ L (μm)	Finger	Multiplier
M_1_, M_2_	1/4	1	2
M_3_, M_4_, M_5_, M_6_, M_9_, M_10_	2/4	1	2
M_7_, M_8_, M_11_, M_12_	2/4	1	8

**Table 2:** The parameters of the CMOS transistors in the operational amplifier and performance indicators of the operational amplifier.

CMOS Parameters			
Instance Name	W (μm)/ L (μm)	Finger	Multiplier
M_1_, M_2_	1/0.5	1	2
M_3_, M_4_, M_5_, M_6_	1/1	1	2
M_7_, M_9_	2/1	1	2
M_8_	2/1	1	4
M_10_	2/1	1	2
Circuit Performance Parameters			
Gain (G)		42 dB	
3 dB Bandwidth (BW)		615 kHz	
Phase margin (PM)		56 deg	

**Table 3:** The parameters of the CMOS transistors in the comparator and performance indicators of the comparator.

CMOS Parameters			
Instance Name	W (μm)/ L (μm)	Finger	Multiplier
M_1_, M_2_, M_3_, M_4_, M_5_	2/4	1	2
M_6_	4/4	1	2
M_7_	4/4	1	4
M_8_	4/4	1	1
Circuit Performance Parameters			
Gain (G)		81.4 dB	
3 dB Bandwidth (BW)		43.1 kHz	

**Table 4:** Comparison of performance parameters for CMOS temperature sensors.

Parameters	[19]	[20]	[21]	[22]	[23]	[24]	[25]	[26]	This Work
Technology	160 nm	180 nm	180 nm	180 nm	130 nm	130 nm	130 nm	16 nm	180 nm
type	CMOS	CMOS	CMOS	CMOS	CMOS	CMOS	CMOS	FinFET	CMOS
Area (mm^2^)	0.15	0.13	0.45	0.09	0.07	0.0014	0.29	0.0126	0.059
Supply (V)	1.8	1.8/3.3	0.6	1.2	0.95	0.85	2–3.6	1.8	1.8
Temp. range (°C)	−40–180	−50–150	0–100	0–100	0–80	−60–40	−40–125	−50–150	0–100
Inaccuracy (°C)	±0.2	±0.8	+0.62/−1.33	+1.5/−1.4	+0.44/−0.4	±2	±0.47	±2	+0.65/−0.49
Calibration	1-point	1-point	2-point	2-point	2-point	2-point	1-point	0-point	2-point
Resolution (°C)	0.023	0.04	0.1	0.3	0.1	0.5	0.016	0.38	0.003
Power (μW)	9.7	45.7	0.075	0.071	0.196	0.15	313.5	1210	32.9
Conversion rate (ms)	20	10.24	254	30	59	1000	5.12	0.27	22.75
Resolution *FOM (pJ/K^2^)	103	748	190	190	120	37500	411	47175	6.7

$\mathrm{FOM}\left[\mathrm{pJ}/K^{2}\right]=\mathrm{Energy}/\mathrm{conversion}\times {\left(\mathrm{Resolution}\right)}^{2}$ [22].

## Data Availability

Not applicable.
